# Exploring the contribution of mammary-derived serotonin on liver and pancreas metabolism during lactation

**DOI:** 10.1371/journal.pone.0304910

**Published:** 2024-06-05

**Authors:** Sena L. Field, Everardo Anta Galvan, Laura L. Hernandez, Jimena Laporta

**Affiliations:** Department of Animal and Dairy Sciences, University of Wisconsin-Madison, Madison, WI, United States of America; Johnson & Johnson MedTech, UNITED STATES

## Abstract

During lactation, the murine mammary gland is responsible for a significant increase in circulating serotonin. However, the role of mammary-derived serotonin in energy homeostasis during lactation is unclear. To investigate this, we utilized C57/BL6J mice with a lactation and mammary-specific deletion of the gene coding for the rate-limiting enzyme in serotonin synthesis (*TPH1*, Wap-Cre x TPH1^FL/FL^) to understand the metabolic contributions of mammary-derived serotonin during lactation. Circulating serotonin was reduced by approximately 50% throughout lactation in Wap-Cre x TPH1^FL/FL^ mice compared to wild-type mice (TPH1^FL/FL^), with mammary gland and liver serotonin content reduced on L21. The Wap-Cre x TPH1^FL/FL^ mice had less serotonin and insulin immunostaining in the pancreatic islets on L21, resulting in reduced circulating insulin but no changes in glucose. The mammary glands of Wap-Cre x TPH1^FL/FL^ mice had larger mammary alveolar areas, with fewer and smaller intra-lobular adipocytes, and increased expression of milk protein genes (e.g., WAP, CSN2, LALBA) compared to TPH1^FL/FL^ mice. No changes in feed intake, body composition, or estimated milk yield were observed between groups. Taken together, mammary-derived serotonin appears to contribute to the pancreas-mammary cross-talk during lactation with potential implications in the regulation of insulin homeostasis.

## Introduction

Serotonin (5-hydroxytryptamine) is an evolutionarily conserved monoamine with notable roles in cell signaling across many species in countless organ systems [1]. Serotonin is derived from the essential amino acid L-tryptophan in a two-step pathway. First, L-tryptophan is converted by tryptophan hydroxylase (TPH, rate-limiting step) to 5-Hydroxy-L-Tryptophan (5-HTP, serotonin precursor). Finally, 5-HTP is converted to serotonin via a ubiquitous aromatic amino acid decarboxylase enzyme. Two independent serotonergic systems, non-neuronal and neuronal, are regulated by distinct genes encoding the TPH enzyme (TPH1 and TPH2, respectively) [2]. Neuronal serotonin has been explored extensively for its role as a neurotransmitter involved in modulating behavior and depression [3]. Over the last few decades, increasing evidence supports the role of non-neuronal serotonin (i.e., peripheral to the brain) as a regulator of biological processes, including lactation homeostasis and energy metabolism. Global ablation of *TPH1* in mice has served as a valuable model for understanding systemic autocrine/paracrine serotonergic networks [4]. Yet, the tissue-specific contributions of serotonin warrant further investigation.

Serotonin is a local regulator of mammary gland homeostasis and has been shown to act as a feedback inhibitor of lactation [5, 6]. Human, murine, and bovine *in vitro* and *in vivo* models demonstrated that serotonin regulates mammary epithelial cell (MEC) tight junctions, alveolar milk volume, and milk protein gene expression by signaling in MEC. This regulation is exerted primarily through the serotonin receptor 7 (5-HTR7) [5, 7, 8]. During lactation, serotonin plays a role in the mobilization of calcium from bone through the regulation of parathyroid hormone-related protein (PTHLH) secretion [9, 10].

It is also well documented that peripheral serotonin acts as an endocrine factor in a variety of tissues regulating energy homeostasis [11, 12]. In the murine pancreas, specifically the β-cells within the islet of Langerhans, serotonin is co-secreted with insulin, and studies have shown that loss of serotonin in the β-cell impairs insulin secretion [13, 14]. During pregnancy, serotonin signals through serotonin receptor 2B (5-HTR2B) in the maternal islets, acting as a key regulator of β-cell mass and expansion [15]. RNA-sequencing data from isolated islets of lactating female mice revealed serotonin biosynthesis is activated during lactation relative to gestation, and this increase is accompanied by an expansion in β-cell mass [16]. In fasted mice, serotonin signals through the 5-HTR2B in hepatocytes inhibit glucose uptake and stimulate gluconeogenesis, maintaining blood glucose levels [17]. Also, late-lactation dairy cows with artificially elevated circulating serotonin concentrations experience greater glucose concentrations and a highly dynamic and dose-dependent expression of liver gluconeogenic genes [18].

Utilizing the murine whey acidic protein (*WAP*)-Cre recombinase, deletion of *TPH1* in MEC is induced during late pregnancy and lactation, which allowed the exploration of mammary-specific contributions of serotonin to the maintenance of energy homeostasis during lactation [19, 20]. Using this mouse model, Weaver and colleagues showed that mammary gland-derived serotonin contributes to 44% of peripheral blood serotonin during lactation, yet the peripheral implications of this reduction were unexplored [21]. The objectives of this study were to investigate the consequences of mammary-specific disruption of serotonin on liver and pancreas glucose and insulin secretion during lactation. We hypothesized that mammary-derived serotonin acts in an endocrine manner via its G-protein coupled receptors to regulate insulin and glucose synthesis in the liver and pancreatic tissue during lactation.

## Materials and methods

### Animals

All procedures were conducted under protocols approved by the College of Agriculture and Life Sciences Institutional Animal Care and Use Committee at the University of Wisconsin-Madison (#A005789-03). C57BL/6 mice were held in a controlled environmental facility for animal research in the Department of Biochemistry at the University of Wisconsin- Madison at 50–60% humidity, 25°C, a 12 h light: 12 h dark cycle with access to ad libitum food and water. Primiparous mice (6–8 weeks old) were used to disrupt *TPH1*, a rate-limiting enzyme in serotonin synthesis [20]. Homozygous female mice with the *TPH1* gene flanked with two loxP sites (TPH1^FL/FL^) globally were crossed with C57BL/6 mice that express Cre recombinase under the control of the *WAP* promoter (Wap-Cre × TPH1^FL/FL^, n = 5) [19]. All genotypes were confirmed using protocols described in [21]. At approximately pregnancy day 16 in pregnant mice, WAP synthesis initiates the expression of Cre recombinase within mammary epithelial cells, subsequently triggering the gene disruption of *TPH1* creating the conditional knockout [21]. Primiparous female mice homozygous for loxP sites (TPH1^FL/FL^) flanking *TPH1* were used as the control group (n = 5).

#### Data and sample collection

Mice were enrolled in the study on the day of conception, evidenced by a vaginal mucus plug (baseline, P0). Body composition of the dams was measured on P0, E17.5 (late gestation), and lactation day 1 (L1, early lactation), 10 (L10, peak lactation) and 21 (L21, late lactation) using dual-energy x-ray absorptiometry (DEXA) via a PIXImus2 Mouse Densitometer (GE Medical Systems, Madison, WI). Lunar PIXImus software was used to analyze the scans using auto-thresholding to measure total tissue mass (TTM), total fat mass (TFM) and total lean mass (TLM). Feed intakes were recorded daily on P0-P18 and L1-L21. Pup total body weights were recorded daily (L1-L21), and individual body weights were estimated by dividing by the pup litter number.

Milk yield was determined by measuring milk consumption per pup on L10 and L21 using the weigh-suckle-weigh (WSW) method [22, 23]. Briefly, pups were weighed, then separated from their dams at 0800 h for 4 h by relocation in a ventilated pipette tip box placed in their original home cage. Pups were then reunited with their mothers to nurse for 45 mins, then weighed again. The difference in pup weights is calculated as an estimation of milk yield during this 4 h period.

Mice were fasted for 6 h prior to blood collection on P0, L1, L10 and L21. Blood was collected from the submandibular vein using a 5.5 mm lancet on P0, L1 and L10 and from cardiac puncture on L21. Immediately following collection, blood was centrifuged at 1500 x g at 4°C for 20 min for serum isolation and stored at -80°C until further analysis.

On L21, the final day in the mouse lactation cycle, dams were fasted for 6 h and then euthanized via carbon dioxide inhalation, followed by cervical dislocation for tissue collection. The number 3 mammary glands, pancreas, liver, and duodenum were snap-frozen in liquid nitrogen and stored at -80°C until RNA or protein isolation. For histological analyses, number 4 mammary glands, pancreas and liver tissue were fixed in 10% formalin overnight, then transferred to PBS and stored at 4°C until embedding in paraffin.

### Blood analyses

Glucose concentrations were quantified in serum samples in duplicate using an assay kit (Crystal Chem, #81692), per manufacturer’s instructions. Insulin concentrations were quantified in serum samples in duplicate using the ELISA kit (Crystal Chem, #90080), per manufacturer’s instructions. Serotonin concentrations were quantified in serum samples diluted 1:150 in duplicate using a serotonin enzyme immunoassay kit (Immunotech, Beckman Coulter, #IM1749), per the manufacturer’s instructions.

### Tissue serotonin concentrations

Protein was extracted from mammary, pancreas, liver, and duodenum tissue using radioimmunoprecipitation assay buffer with 10 μm/mL of Halt Protease and Phosphatase Inhibitor Cocktail (Thermo Scientific, #23227). Protein concentrations were determined using bicinchoninic acid assay (Pierce Chemicals, #23227). All tissues were diluted 1:20 and assayed in duplicate. Tissue serotonin concentrations were quantified using 50 μg of protein per sample using a serotonin enzyme immunoassay kit (Immunotech, Beckman Coulter, #IM1749), per the manufacturer’s instructions.

### Tissue gene expression

Total RNA was extracted from mammary and liver tissue using TRIzol and the Invitrogen PureLink RNA Mini Kit (Thermo Fisher, #12183018A) per the manufacturer’s instructions. RNA quantity and quality were measured using the Implen Nanophotometer and one μg of RNA was reverse transcribed using iScript Reverse Transcription Supermix (Bio-Rad, Hercules, CA; #1708841). Real time- qPCR was performed using CFX96 Real-Time PCR detection System (Bio-Rad) with reaction mixtures and cycling conditions performed as previously described [24]. The geometric mean of three housekeeping genes, S15, Rsp9, and K8 for mammary and Rsp9 and S15 for liver tissue, were calculated to normalize gene expression. Primer sequences can be found in Supplemental S1 Table. All primer sequences were designed to span exon-exon junctions and an optimal annealing temperature of 60°C, to minimize the potential of amplifying genomic DNA, using Primer3 software with sequences obtained from GenBank (http://www.ncbi.nlm.nih.gov/). All primer pairs displayed melting curves with a single peak, indicative of a pure, single amplicon, confirming the specificity of the primers.

### Tissue histology and immunofluorescence

Hematoxylin and eosin (H&E) staining was performed on mammary, liver and pancreas tissue according to standard staining procedures. Four photomicrographs were obtained per animal section at 20x magnification with the BZ-X800 Keyence microscope. Alveoli area, number and adipocyte total area and number from mammary, islet of Langerhans area from pancreas and hepatocyte number from liver were quantified. Alveoli and islet of Langerhans area were quantified using ImageJ software [25]. Fields were selected at random avoiding overlapping and edges of the section.

Pancreas sections were deparaffinized and incubated with serotonin (Immunostar, #20080, 1:5000) and insulin (DAKO, #A0564, 1:500) primary antibodies. Secondary antibodies were diluted 1:500 and incubated 1h with either Alexa Fluor 555 Goat Anti-Rabbit (#A21429) or Alexa Fluor 488 Goat Anti-Guinea Pig (#A11073) and nuclei visualized staining 4′,6′-diamidino-2-phenylindole (DAPI). The Keyence microscope was used to visualize islet microstructure and fluorescence by capturing four photomicrographs in one tissue section (20× magnification) per mouse to quantify serotonin and insulin intensity. The BZ-X800 Analyzer software was used to quantify intensity of the serotonin (red) and insulin (green). Specifically, the target area threshold was set at 14 and 20 for serotonin and insulin, respectively, to quantify sum target integration (i.e., the value of multiplying the extracted part area by the intensity of the component before binarization).

### Statistical analysis

Data were analyzed by ANOVA using the MIXED procedure of SAS v. 9.4 (SAS Institute Inc, Cary, NC). Models included the fixed effect of genotype (TPH1^FL/FL^ or Wap-Cre x TPH1^FL/FL^), time (days, as repeated measure), and their interaction. Residuals were tested for normality and homogeneity of variance, and the first-order autoregressive covariance structure (AR-1) was used as the covariance structure for repeated measures. If residuals deviated from normality, data were transformed following Box-Cox lambda and back transformed for visual representation. Unless otherwise stated, data are presented as the least squares means ± standard error of the mean (LSM ± SEM). Gene expression data is displayed as model estimates (ΔΔCt). Statistical significance was declared at *P* ≤ 0.05 and tendencies at 0.05 < *P* ≤ 0.10.

## Results

### Body weight, composition, milk consumption, and feed intake

To investigate if mammary-derived serotonin regulates body composition, dual x-ray absorptiometry (DEXA) scans were performed throughout gestation and lactation to measure total tissue, fat, lean mass, and fat mass percentage. No differences were detected in Wap-Cre x TPH1^FL/FL^, compared to TPH1^FL/FL^ dams for all variables analyzed (**Table 1**). Utilizing the WSW method, milk yield was determined by measuring milk consumption per pup on L10 and L21. No differences were observed between Wap-Cre x TPH1^FL/FL^ and TPH1^FL/FL^ dams S1A Fig, *P* ≥ 0.96). Dam weights were assessed daily throughout gestation (P0-P18) and lactation (L1-L21), and pup weights were taken for the first 21 days of life. No differences were detected between dams (S1B Fig, *P* = 0.75) or pups (S1C Fig, *P* = 0.87) born to Wap-Cre x TPH1^FL/FL^ and TPH1^FL^ dams. Daily feed intakes were measured throughout gestation and lactation, and no differences were found between Wap-Cre x TPH1^FL/FL^ and TPH1^FL^ dams (S1D Fig, *P* = 0.79). Litter size was recorded throughout lactation and no differences were found between Wap-Cre x TPH1^FL/FL^ and TPH1^FL^ dams (S1E Fig, *P* = 0.78).

**Table 1:** Body composition data from TPH1^FL/FL^ and Wap-Cre x TPH1^FL/FL^ C57BL/6 mice using dual x-ray absorptiometry (DEXA) on days P0, E17.5, L1, L10 and L21. Data are represented as the LSM ± SEM.

			*P*-value		
	TPH1^FL/FL^	Wap-Cre x TPH1^FL/FL^	Genotype	Day	Genotype x Day
Body Composition					
Total Tissue Mass, g	22.81 ± 0.45	23.22 ± 0.45	0.53	< 0.001	0.88
Total Fat Mass, g	3.92 ± 0.09	3.93 ± 0.09	0.92	< 0.001	0.68
Total Lean Mass, g	18.88 ± 0.42	19.29 ± 0.42	0.5	< 0.001	0.92
Total Fat Mass, %	17.68 ± 0.55	17.35 ± 0.55	0.68	< 0.001	0.82

### Validation of the mammary gland *TPH1* knock-out model

**Fig 1:**
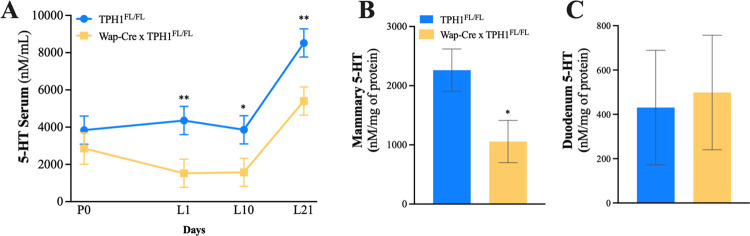
Mammary-specific disruption of *TPH1* reduces circulating and mammary serotonin by 1-fold. Model validation of control dams (TPH1^FL/FL^, n = 5) and dams with mammary gland deletion of *TPH1* (Wap-Cre x TPH1^FL/FL^, n = 5) first lactation mice had (**A**) serum collected on pregnancy day 0 (P0) and during lactation (L1, L10 and L21) to measure circulating serotonin concentrations. On L21, mice were euthanized to harvest organs to measure (**B**) mammary gland and (**C**) duodenum serotonin concentrations. Data is presented as least squares means (LSM) ± standard error of the mean (SEM). Significance declared at (*) *P* ≤ 0.05.

Mice with mammary-specific disruption of *TPH1* have previously been developed by using the WAP-*Cre* Cre-lox system, which is activated during late gestation through lactation [21]. In the present study, circulating serum serotonin concentrations were reduced throughout lactation (L1-L21) in Wap-Cre x TPH1^FL/FL^ dams, compared to TPH1^FL/FL^ (**Fig 1A**, *P* = 0.008). Serotonin content in the mammary gland at L21 was reduced by 53.3% in Wap-Cre x TPH1^FL/FL^ dams, compared to TPH1^FL/FL^ dams (**Fig 1B**, *P* = 0.04). No differences were observed in serotonin content in the duodenum between the two groups (**Fig 1C**, *P* = 0.24), indicating that the disruption was local to the gland tissue.

### Mammary microstructure and gene expression

**Fig 2:**
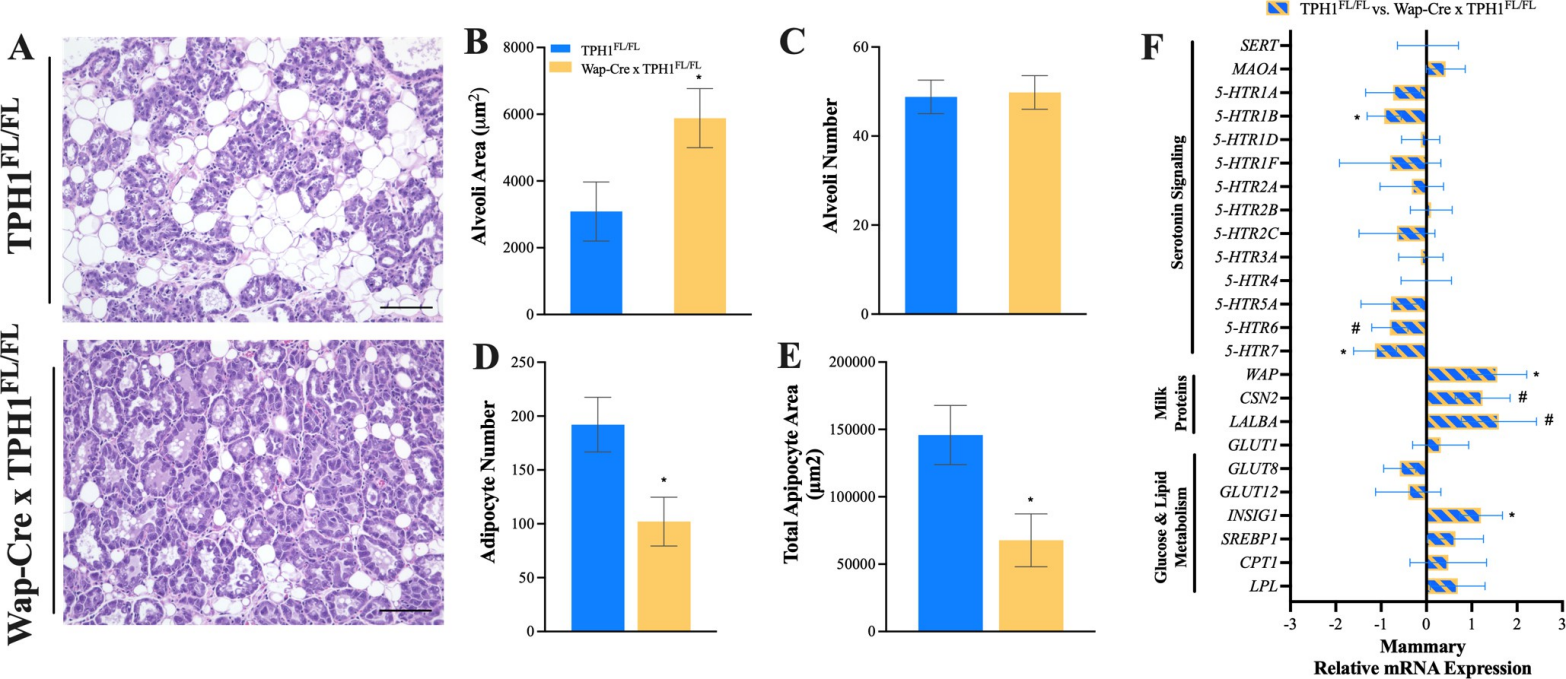
Mammary-specific disruption of *TPH1* averts involution. Control dams (TPH1^FL/FL^, n = 5) and dams with mammary gland deletion of *TPH1* (Wap-Cre x TPH1^FL/FL^, n = 5) were euthanized on L21 for histological and transcriptional evaluation of the mammary gland. (**A**) Hematoxylin and eosin (H&E) staining at 20 × magnification to quantify (**B**) alveoli area (**C**) alveoli number (**D**) adipocyte number and (**E**) total adipocyte area. (**F**) Mammary gland gene expression of serotonin synthesis and metabolism (*TPH1*, *SERT*, *5-HTR1A*, *-1B*, *-1D*, *-1F*, *-2A*, *-2B*, *-2C*, *-3A*, *-3B*, *-4*, *-5A*, *-6*, and *-7)*, glucose transporters (*GLUT1*, *-8* and *12*) and lipid metabolism genes (*INSIG1*, *SREBP1*, *CPT1* and *LPL*) were measured. Positive or negative relative mRNA expression (ΔΔCt) indicates gene upregulation and downregulation of TPH1^FL^ × Wap^Cre^ mice relative to TPH1^FL^, respectively. Scale bar = 100 μm. Data is presented least squares means (LSM) ± standard error of the mean (SEM). Significance declared at (*) *P* ≤ 0.05 and (#) denotes a statistical tendency at 0.05 < *P* ≤ 0.10.

Mammary glands were harvested on L21 for H&E staining and gene expression analysis (**Fig 2**). Alveoli number was similar between groups (**Fig 2B**, *P* = 0.85), and alveoli area was greater in Wap-Cre x TPH1^FL/FL^ dams, compared to TPH1^FL/FL^ (**Fig 2C**, *P* = 0.05). The number of inter- and intra-lobular adipocytes in the mammary tissue was counted. A reduction in adipocyte number and total adipocyte area in Wap-Cre x TPH1^FL/FL^ dams was observed compared to TPH1^FL/FL^ (**Fig 2D and 2E**, *P* = 0.03).

Genes related to serotonin signaling, glucose, milk and lipid synthesis, and metabolism were measured in the mammary glands harvested at L21. Serotonin receptors (5-HTR) *-1B* and *-7* were downregulated, and *5-HTR6* tended to be downregulated in Wap-Cre x TPH1^FL/FL^ dams, compared to TPH1^FL/FL^ (**Fig 2F**, *P* < 0.08). Insulin-induced gene 1 (*INSIG1*) was upregulated in Wap-Cre x TPH1^FL/FL^ dams, compared to TPH1^FL/FL^ (**Fig 2F**, *P* = 0.03). Whey acidic protein (WAP) was upregulated, and CSN2 and LALBA tended to be upregulated in Wap-Cre x TPH1^FL/FL^ dams, compared to TPH1^FL/FL^ (**Fig 2F**, *P* < 0.02).

### Pancreas islet of Langerhans staining and circulating serum insulin

**Fig 3:**
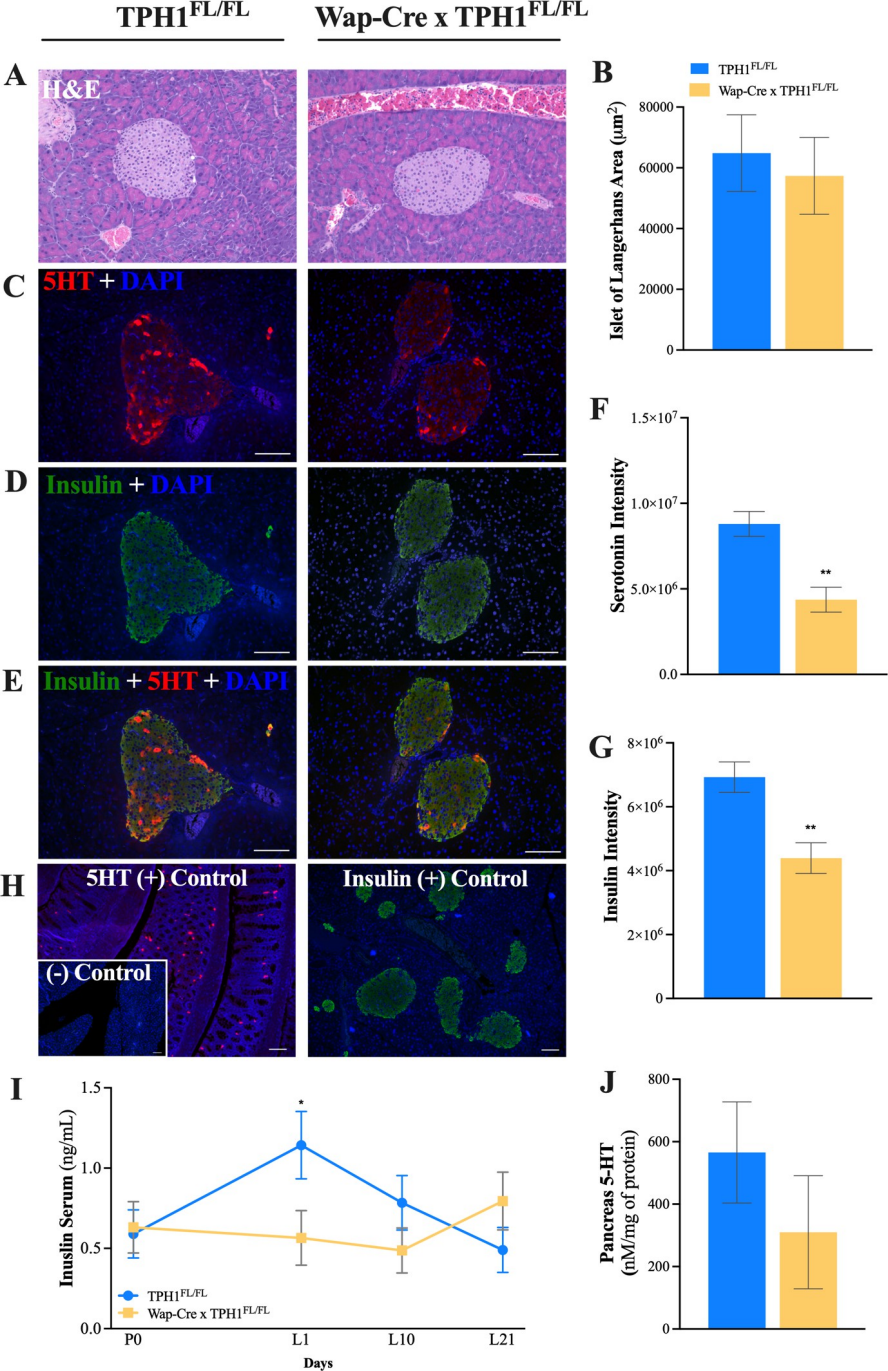
Mammary-specific disruption of *TPH1* reduces pancreatic serotonin and insulin. Control dams (TPH1^FL/FL^, n = 5) and dams with mammary gland deletion of *TPH1* (Wap-Cre x TPH1^FL/FL^, n = 5) were euthanized on L21 for histological evaluation of the pancreas. (**A**) Hematoxylin and eosin (H&E) staining at 20 × magnification to quantify (**B**) islet of Langerhans area. (**C-E**) Immunofluorescence staining in pancreas tissue at 40 × magnification to quantify (**F**) serotonin (5-HT) and (**G**) insulin intensity. (**H**) Positive controls for staining 5-HT and insulin using mouse duodenum and pancreas, respectively. The negative control had no primary antibody applied. (**I**) Serum was collected on pregnancy day 0 (P0) and during lactation (L1, L10 and L21) to measure circulating insulin concentrations and (**J**) pancreas protein 5-HT concentrations were measured at L21. Scale bar = 100 μm. Data is presented least squares means (LSM) ± standard error of the mean (SEM). Significance declared at (*) *P* ≤ 0.05 and (**) *P* ≤ 0.001.

Pancreas tissue was harvested on L21 for H&E microstructure characterization and immunofluorescence staining of serotonin and insulin (**Fig 3A–3H**). The surface area of the Islet of Langerhans was not different between groups (**Fig 3B**, *P* = 0.68). Serotonin and insulin intensity were reduced in the islet of Langerhans of Wap-Cre x TPH1^FL/FL^ dams, compared to TPH1^FL/FL^ (**Fig 3F and 3G**, *P* < 0.005). Circulating serum insulin concentrations were reduced on L1 in Wap-Cre x TPH1^FL/FL^ dams (**Fig 3I**, *P* = 0.08), and no differences were observed in serotonin content in the pancreas tissue (**Fig 3J**, *P* = 0.32). The number of islets analyzed per mouse were similar between genotypes (TPH1^FL/FL^ = 8.6 ± 0.678 islets; Wap-Cre x TPH1^FL/FL^ = 8.4 ± 0.678 islets, P = 0.84).

### Liver microstructure, gene expression, and circulating serum glucose concentrations

**Fig 4:**
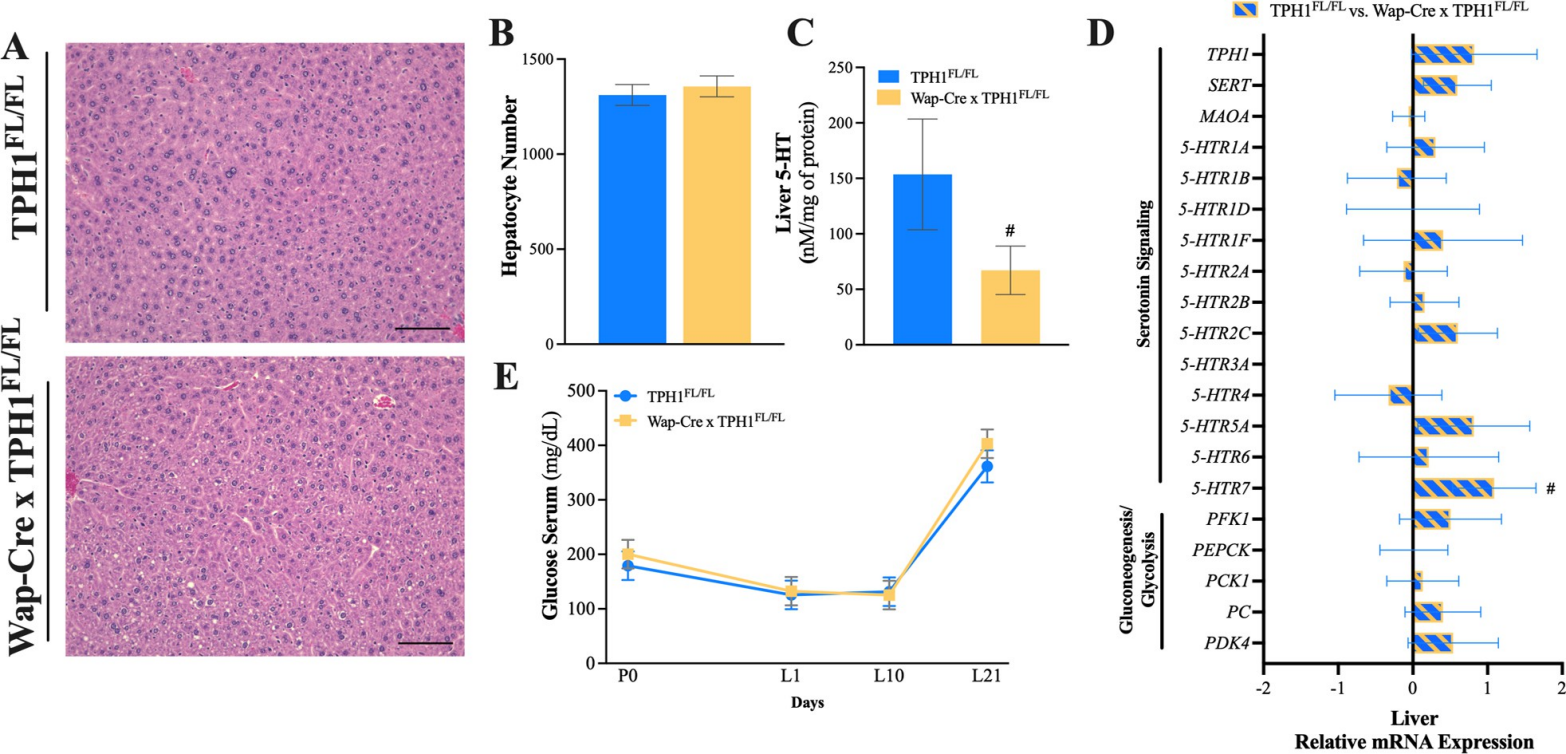
Mammary-specific disruption of *TPH1* tends to reduce liver serotonin concentrations. Control dams (TPH1^FL/FL^, n = 5) and dams with mammary gland deletion of *TPH1* (Wap-Cre x TPH1^FL/FL^, n = 5) were euthanized on L21 for histological and transcriptional evaluation of the liver. (**A**) Hematoxylin and eosin (H&E) staining at 20 × magnification was performed to quantify (**B**) hepatocyte number. (**C**) Liver serotonin (5-HT) concentrations at L21 and (**D**) liver gene expression of serotonin synthesis and metabolism (*TPH1*, *SERT*, *5-HTR1A*, *-1B*, *-1D*, *-1F*, *-2A*, *-2B*, *-2C*, *-3A*, *-3B*, *-4*, *-5A*, *-6*, and *-7)* and glucose metabolism (*PFK1*, *PEPCK*, *PCK1*, *PC* and *PDK4*) genes were measured. (**E**) Serum was collected on pregnancy day 0 (P0) and during lactation (L1, L10, and L21) to measure circulating glucose concentrations. Positive or negative relative mRNA expression (ΔΔCt) indicates gene upregulation and downregulation of Wap-Cre x TPH1^FL/FL^ mice relative to TPH1^FL/FL^, respectively. Scale bar = 100 μm. Data is presented as least squares means (LSM) ± standard error of the mean (SEM). Significance is declared at (*) *P* ≤ 0.05, and (#) denotes a statistical tendency at 0.05 < *P* ≤ 0.10.

Liver tissue was harvested on L21 for H&E staining and gene expression analysis. The number of hepatocytes was similar between groups (**Fig 2B**, *P* = 0.57). Liver tissue serotonin content tended to be reduced (**Fig 4C**, *P* = 0.10), and *5-HTR7* gene expression tended to be upregulated in Wap-Cre x TPH1^FL/FL^ dams, compared to TPH1^FL/FL^ (**Fig 4D**, *P* = 0.08). Circulation glucose concentrations were similar between groups (**Fig 4E**, *P* = 0.49).

## Discussion

The energy requirements driven by lactogenesis induce changes in maternal metabolism for the partitioning of nutrients to both the dam and offspring. Serotonin is known to regulate the mammary gland-bone cross-talk during lactation to aid in calcium homeostasis and plays an important role in alveolar homeostasis [10, 26, 27]. Steps towards deciphering the mammary-specific contributions of serotonin to the peripheral serotonergic pool during lactation have been possible through the development of a mammary epithelial cell conditional knockout of *TPH1*. Yet, we currently lack an understanding of the endocrine role of mammary-derived serotonin in the cross-talk with metabolic peripheral tissues during lactation. The present study elucidates the consequences of ablating mammary gland serotonin’s contribution to the systemic serotonin pool on mammary homeostasis and liver and pancreas metabolism.

In the present study, Wap-Cre x TPH1^FL/FL^ dams had a one-fold reduction in circulating serotonin concentrations throughout lactation and a reduction in mammary serotonin tissue content on L21. This data is in accordance with our groups’ previous findings, where dams of the same genotype had a reduction of circulating serotonin and mammary serotonin tissue content on L10 [21]. Herein, mammary-specific disruption of *TPH1* did not alter serotonin duodenum tissue content, which is also consistent with previous findings and confirms the alterations in circulating serotonin concentrations are a direct consequence of the *TPH1* disruption in the mammary gland driven by the WAP-Cre recombinase.

Recent studies have brought to light an interesting correlation between serotonin and obesity. Increased duodenum *TPH1* expression and peripheral serotonin levels are shown to be positively correlated to body mass index [28]. In addition, an adipose tissue *TPH1* KO revealed a resistance to high-fat diet-induced obesity and a reduction in adipose tissue lipid accumulation [29]. In the present study, mammary-specific disruption of TPH1 did not alter body composition (i.e., total fat, tissue, and lean mass), suggesting mammary-derived serotonin does not regulate peripheral adipocyte metabolism at the phenotypic level during lactation. Since peripheral and central serotonin are known to regulate appetite, we sought to measure maternal daily feed intakes, as well as dam and pup body weights [30, 31]. Herein, mammary-specific *TPH1* disruption did not alter maternal feed intakes, subsequently not modifying dam and pup weights between genotypes.

A hallmark of murine mammary gland involution is the disappearance of alveolar structures and regeneration of adipocytes, which “refill” the mammary gland mass, although the exact molecular mechanisms for mammary adipocyte remodeling during involution are beginning to be elucidated [32, 33]. In mammals, serotonin downregulates milk protein genes, and degrades tight junctions between mammary epithelial cells through the serotonin receptor 7 (5-HTR7) [5, 8]. Evidence also suggests serotonin receptor -1B (5-HTR1B) regulates milk protein status in primary bovine mammary epithelial cells [34]. In our study, mammary gland microstructure was altered in Wap-Cre x TPH1^FL/FL^ dams on L21 (i.e., the final day of lactation), exhibiting an increase in alveoli area, and a reduction in adipocyte number and total adipocyte area. Simultaneously, serotonin receptors -*1B*, -*6*, and *-7* were downregulated, and milk protein genes (i.e., *WAP*, *CSN2*, and *LALBA*) were upregulated in dams with a mammary disruption of *TPH1*. This data suggests the local reduction in mammary serotonin synthetic capacity alters the signaling pathways required to initiate mammary involution, thus potentially delaying its onset. This decoupling of serotonin signaling in the mammary gland results in lower autocrine serotonin signaling in the mammary gland near the end of lactation. Despite histological differences in the mammary microstructure, no differences were observed in dam milk yield on L10 and L21.

Previous studies in mice have reported increased serum serotonin levels during progressing lactation [21, 35]. Serotonin synthesis by pancreatic islet cells also increases in lactating mice pancreatic islets compared to post-partum non-lactating counterparts [16]. More specifically, expression of *TPH1* and *TPH2* was enhanced in the islets of lactating mice, supported by an increase in positive serotonin-stained cells. Moreover, precise double staining quantification of insulin and serotonin-positive cells within the islets revealed all serotonin-positive cells were also stained for insulin, implying a role for serotonin in β-cell homeostasis during lactation [16]. In the present study, a reduction in mammary gland serotonin led to reduced serotonin and insulin immunostaining within the islets on L21 without altering the islet area. Serum insulin concentrations were also reduced on L1 in Wap-Cre x TPH1^FL/FL^ mice during the fasted state, although no differences were observed in glucose concentrations. This data suggests mammary-derived serotonin works in an endocrine manner to regulate insulin synthesis and secretion in peripheral tissues at least in late lactation. However, further research is needed to delineate the mechanism of action mammary serotonin plays in the endocrine regulation of insulin synthesis within the islets during lactation. Understanding the cross-talk between mammary-derived serotonin and metabolic tissues might further provide a role for serotonin in metabolic disorders, potentially regulating glucose intolerance.

The liver expresses the machinery necessary to synthesize, signal and metabolize serotonin which is known to regulate a variety of functions in liver biology such as tissue regeneration and hepatic blood flow [36, 37]. It is also well accepted that the mammary gland and liver work as a functional unit during lactation by increasing the metabolic output needed to meet the nutritional demands [38]. Several lines of evidence in multiple species have indicated the liver is the primary source of glucose during lactation, although the regulation for this exact mechanism remains unknown [39, 40]. Additionally, intestine-derived serotonin acts through the serotonin receptor 2B in the liver to stimulate gluconeogenesis [17]. We postulate that mammary-derived serotonin could play a mediating role in the regulation of energy demands of the liver. To our surprise, dams with the mammary-specific disruption of *TPH1* had a reduction in serotonincontent in the liver, implying that mammary-derived serotonin during lactation might act in an endocrine manner to impact liver serotonin synthesis. No alterations in gluconeogenic enzyme transcripts, serum glucose concentrations or hepatocyte number were observed, suggesting this privation of liver serotonin content does not impact glucose homeostasis or cellular turnover near the end of lactation. In murine hepatocytes, 5-HTR7 couples to c-AMP and was reported to trigger elevated Ki67 expression and increased IGF-1 secretion, although its role in an in vivo lactating model is unknown [41]. In the present study, the 5-HTR7 liver mRNA expression tended to be upregulated in dams with the mammary-specific disruption of *TPH1*. Further research is required to examine the underpinnings of serotonin function in liver biology during lactation.

## Conclusions

This is the first in vivo study exploring the potential cross-talk of mammary-specific serotonin with peripheral metabolic tissues during established lactation. Mammary-specific TPH1 knockout mice had a 40–50% reduction in mammary gland and liver serotonin content, a 50% reduction in serum serotonin concentration throughout lactation, and reduced serum insulin without major changes in circulating glucose. Body growth and composition characteristics of the dam and pup growth were unaltered by mammary-specific serotonin deletion. The reduction of mammary serotonin appeared to delay the natural involution progression of the glandular tissue in response to the self-weaning of neonates in late lactation. Additionally, the lower levels of insulin and serotonin in the bloodstream, with insulin staining in pancreatic islet cells suggest mammary-derived serotonin may act endocranially to regulate hormone production in the pancreas. Further research is needed to investigate how serotonin and insulin co-secretion and synthesis in the pancreas contribute to determining energy reserves, such as adipose tissue mobilization, during peak lactation at both the extracellular and intracellular levels.

## Supporting information

S1 Table Primer sequences utilized for real-time PCR analysis of genes involved in serotonin synthesis and metabolism, milk proteins, lipid metabolism, and glycolysis/gluconeogenesis enzymes in the mammary gland or liver tissue lactating female C57BL/6 mice. All primer sequences were designed to span exon-exon junctions to minimize the potential of amplifying genomic DNA, using Primer3 software with sequences obtained from GenBank (http://www.ncbi.nlm.nih.gov/). All primer pairs displayed melting curves with a single peak, indicative of a pure, single amplicon, confirming the specificity of the primers.
(DOCX)

S1 Fig Control dams (TPH1^FL/FL^, n = 5) and dams with mammary gland deletion of *TPH1* (Wap-Cre x TPH1^FL/FL^, n = 5) first lactation mice had (**A**) milk yield measured on L10 and L21, (**B**) dam body weights measured daily throughout pregnancy (P0-P18) and lactation (L1-L21), (**C**) pup body weights measured daily on L1-L21 and (**D**) feed intakes recorded daily on P0-P18 and L1-L21. (E) Litter size of pups born to TPH1^FL/FL^ and Wap-Cre x TPH1^FL/FL^ dams. Data is presented as least squares means (LSM) ± standard error of the mean (SEM).
(TIF)

S1 File (XLSX)
