# Supplementary material for: Exploring the contribution of mammary-derived serotonin on liver and pancreas metabolism during lactation
Source: PLoS One. 2024 Jun 5;19(6):e0304910. doi: 10.1371/journal.pone.0304910 (PMC11152252; doi:10.1371/journal.pone.0304910)
Supplement: S1 Table — All primer sequences were designed to span exon-exon junctions to minimize the potential of amplifying genomic DNA, using Primer3 software with sequences obtained from GenBank (http://www.ncbi.nlm.nih.gov/). All primer pairs displayed melting curves with a single peak, indicative of a pure, single amplicon, confirming the specificity of the primers. (DOCX) [file pone.0304910.s001.docx]

**Supplemental Table S1.** Primer sequences utilized for real-time PCR analysis of genes involved in serotonin synthesis and metabolism, milk proteins, lipid metabolism, and glycolysis/gluconeogenesis enzymes in the mammary gland or liver tissue lactating female C57BL/6 mice. All primer sequences were designed to span exon-exon junctions to minimize the potential of amplifying genomic DNA, using Primer3 software with sequences obtained from GenBank (http://www.ncbi.nlm.nih.gov/). All primer pairs displayed melting curves with a single peak, indicative of a pure, single amplicon, confirming the specificity of the primers.

| **Gene** | **Forward Primer (5' --> 3')** | **Reverse Primer (3' --> 5')** | | **NCBI Reference** |
| --- | --- | --- | --- | --- |
| 5-HTR1A | AACTCCAAAGGGCACCTTCC | CACTGTCTTCCTCTCACGGG | | [NM_008308.5](https://www.ncbi.nlm.nih.gov/entrez/viewer.fcgi?db=nucleotide&id=2312957254) |
| 5-HTR1B | CCTGCTGGTTGCTTTGTTGG | CGATCAGGTAGTTAGCCGGG | | [NM_001410237.1](https://www.ncbi.nlm.nih.gov/entrez/viewer.fcgi?db=nucleotide&id=2280436476) |
| 5-HTR1D | TGTCTCCTCCAAACCAGTCC | ACGAGCGAGATTCTGAGAGC | | [NM_001421107.1](https://www.ncbi.nlm.nih.gov/entrez/viewer.fcgi?db=nucleotide&id=2502273150) |
| 5-HTR1F | TCTGGTATCCCTCACTCTGT | CGCTCTCCAAGAAGACTTGA | | [NM_008310.3](https://www.ncbi.nlm.nih.gov/entrez/viewer.fcgi?db=nucleotide&id=162287119) |
| 5-HTR2A | AGGCAAGTCACAGGATAGCG | GCAATGACGGCATTCTAGCC | | [NM_172812.3](https://www.ncbi.nlm.nih.gov/entrez/viewer.fcgi?db=nucleotide&id=1377224679) |
| 5-HTR2B | CAATCATCCTCCTCGATACCC | GAAGCCATCAGATCTACTTTAGCC | | [NM_008311.3](https://www.ncbi.nlm.nih.gov/entrez/viewer.fcgi?db=nucleotide&id=1453799448) |
| 5-HTR2C | CGCTTAGAATAGTGTAGTTAGTTAGGG | GGCTGGAATTGTTTTACTACCC | | [NM_001411391.1](https://www.ncbi.nlm.nih.gov/entrez/viewer.fcgi?db=nucleotide&id=2289442664) |
| 5-HTR3A | TCTTCCAGCCATGGGAAACC | CGGATGGAGGATAGCTCTTGC | | [NM_013561.2](https://www.ncbi.nlm.nih.gov/entrez/viewer.fcgi?db=nucleotide&id=153792455) |
| 5-HTR4 | GGTTCTGCGCTAAAGGTGG | CTGGGGTTCTGATCTCCTGG | | [NM_008313.4](https://www.ncbi.nlm.nih.gov/nucleotide/2452958332?from=61657793&to=61657896&report=gbwithparts) |
| 5-HTR5A | CTTTACTGCCTATGCTCAGCC | TACTCAGCATACCAGGGTGG | | [NM_008314.3](https://www.ncbi.nlm.nih.gov/entrez/viewer.fcgi?db=nucleotide&id=2496840160) |
| 5-HTR6 | GGACCTGATGGTGGGATTGG | AGAGGTTGAGAATGGAGGCG | | [NM_001377096.1](https://www.ncbi.nlm.nih.gov/entrez/viewer.fcgi?db=nucleotide&id=1783658112) |
| 5-HTR7 | ATCAACCGGAAGCTCTCTGC | CACAGTGGTCACAGTTTTGTAGC | | [NM_008315.3](https://www.ncbi.nlm.nih.gov/entrez/viewer.fcgi?db=nucleotide&id=1337002813) |
| Alpha- Lac | CTGCCTCTGAGCCTTGTACC | GTAAAACCCCCATCGAGACC | | [XM_006520517.2](https://www.ncbi.nlm.nih.gov/entrez/viewer.fcgi?db=nucleotide&id=1907110471) |
| Beta-Casein | GGTGAATCTCATGGGACAGC | GAGATGGTTTGAGCCTGAGC | | [NM_009972.2](https://www.ncbi.nlm.nih.gov/entrez/viewer.fcgi?db=nucleotide&id=553727110) |
| CPT1 | ACTCCTGGAAGAAGAAGTTCA | AGTATCTTTGACAGCTGGGAC | | [NM_013495.2](https://www.ncbi.nlm.nih.gov/entrez/viewer.fcgi?db=nucleotide&id=162287141) |
| GLUT1 | TTCTCTGTCGGCCTCTTTGT | CCAGTTTGGAGAAGCCCATA | | [NM_001424864.1](https://www.ncbi.nlm.nih.gov/entrez/viewer.fcgi?db=nucleotide&id=2592185367) |
| GLUT12 | CCCAGCATGTTTACGTTCCT | GTGGCAGGTCAGGGCTAATA | | [NM_178934.4](https://www.ncbi.nlm.nih.gov/entrez/viewer.fcgi?db=nucleotide&id=226437622) |
| GLUT8 | GGAGATGCTCAGACCCTACG | AATGGGCTGTGACTTGTTCC | | [NM_019488.6](https://www.ncbi.nlm.nih.gov/entrez/viewer.fcgi?db=nucleotide&id=2507987712) |
| INSIG1 | ACACGTGGGACCTAACTTGC | TCTGAAATGACCCGAGAACC | | [NM_153526.5](https://www.ncbi.nlm.nih.gov/entrez/viewer.fcgi?db=nucleotide&id=158631238) |
| K8 | ATCGAGATCACCACCTACCG | AAGCCAGGGCTAGTGAGTCC | | [NM_031170.2](https://www.ncbi.nlm.nih.gov/entrez/viewer.fcgi?db=nucleotide&id=114145560) |
| LPL | CCATGGATGGACGGTAACG | TACAGGGCGGCCACAAGT | | [NM_008509.2](https://www.ncbi.nlm.nih.gov/entrez/viewer.fcgi?db=nucleotide&id=126723005) |
| MAOA | ACAGCAACACAGTGGAGTGG | GGAACATCCTTGGACTCAGG | | [NM_173740.3](https://www.ncbi.nlm.nih.gov/entrez/viewer.fcgi?db=nucleotide&id=255759901) |
| PC | CAACACCTACGGCTTCCCTA | CCGGGTGTAATTCTCTTCCA | [NM_008797.3](https://www.ncbi.nlm.nih.gov/entrez/viewer.fcgi?db=nucleotide&id=251823977) | |
| PCK1 | TGACAGACTCGCCCTATGTG | TGCAGGCACTTGATGAACTC | [NM_011044.3](https://www.ncbi.nlm.nih.gov/entrez/viewer.fcgi?db=nucleotide&id=1380941588) | |
| PDK4 | GGATGGAAGGAATCAAAGCA | CACTGGCTTTTTGAGTGCAA | [NM_013743.2](https://www.ncbi.nlm.nih.gov/entrez/viewer.fcgi?db=nucleotide&id=118130875) | |
| PFK1 | CACTGGGACAACATGACCAG | GCAATGGGATCTGATGACCT | [NM_008826.5](https://www.ncbi.nlm.nih.gov/entrez/viewer.fcgi?db=nucleotide&id=1302188133) | |
| RPS15 | TTGAGAAAGGCCAAAAAGGA | GTTGAAGGTCTTGCCGTTGT | [NM_001310726.1](https://www.ncbi.nlm.nih.gov/entrez/viewer.fcgi?db=nucleotide&id=893702688) | |
| RSP9 | GGAGACCCTTCGAGAAGTCG | GGGGATCCTTCTCGTCTAGC | [NM_001419060.1](https://www.ncbi.nlm.nih.gov/entrez/viewer.fcgi?db=nucleotide&id=2468055741) | |
| SERT | GGGACACTTAAGGAGCGCAT | GGAGTCAAGGTGGGGCTTTT | [NM_010484.2](https://www.ncbi.nlm.nih.gov/entrez/viewer.fcgi?db=nucleotide&id=195972806) | |
| SGLT1 | AGCCTCTCTCTTTGCCAGTA | CGGC CTTGATGTAAATCGGG | [NM_019810.4](https://www.ncbi.nlm.nih.gov/entrez/viewer.fcgi?db=nucleotide&id=261824022) | |
| SREBP1 | CCGACATCGAAGACATGC | CCCAGCATAGGGTGGGTCAA | [NM_001313979.1](https://www.ncbi.nlm.nih.gov/entrez/viewer.fcgi?db=nucleotide&id=928192570) | |
| TPH1 | CCCGGAAATCAAAGCAAAG | CTTCCTTCGCAGTGAGCTG | [NM_001136084.2](https://www.ncbi.nlm.nih.gov/entrez/viewer.fcgi?db=nucleotide&id=444909178) | |
| WAP | TATCATCTGCCAAACCAACG | TAGATTCCAAGGGCAGAAGC | [NM_011709.5](https://www.ncbi.nlm.nih.gov/entrez/viewer.fcgi?db=nucleotide&id=146134456) | |
