# Supplementary figures and images for: Exploring the contribution of mammary-derived serotonin on liver and pancreas metabolism during lactation
Source: PLoS One. 2024 Jun 5;19(6):e0304910. doi: 10.1371/journal.pone.0304910 (PMC11152252; doi:10.1371/journal.pone.0304910)

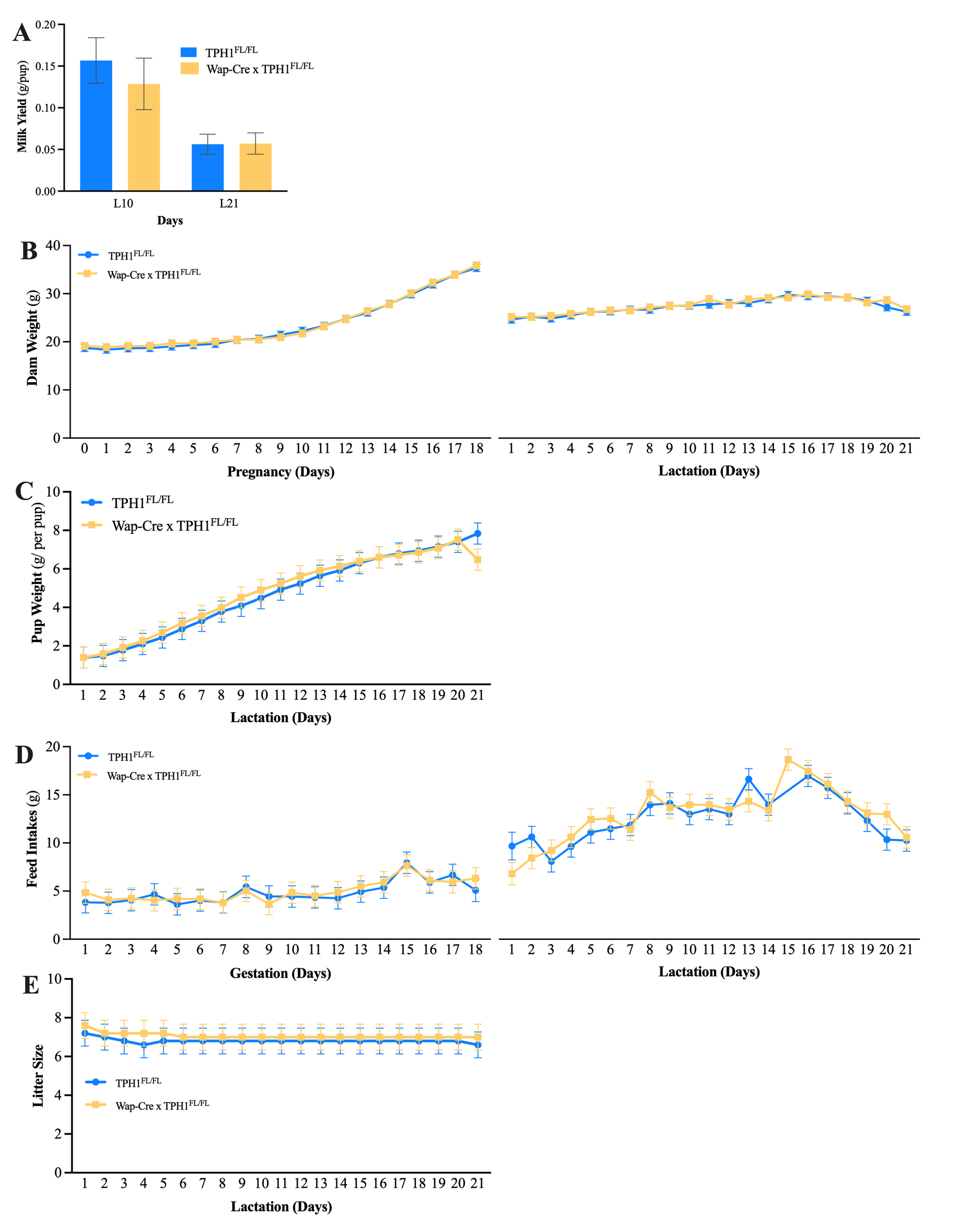

Supplement: S1 Fig — Control dams (TPH1FL/FL, n = 5) and dams with mammary gland deletion of TPH1 (Wap-Cre x TPH1FL/FL, n = 5) first lactation mice had (A) milk yield measured on L10 and L21, (B) dam body weights measured daily throughout pregnancy (P0-P18) and lactation (L1-L21), (C) pup body weights measured daily on L1-L21 and (D) feed intakes recorded daily on P0-P18 and L1-L21. (E) Litter size of pups born to TPH1FL/FL and Wap-Cre x TPH1FL/FL dams. Data is presented as least squares means (LSM) ± standard error of the mean (SEM). (TIF) [file pone.0304910.s002.tif]
